# CrossVit: Enhancing Canopy Monitoring Management Practices in Viticulture

**DOI:** 10.3390/s130607652

**Published:** 2013-06-13

**Authors:** Alessandro Matese, Francesco Primo Vaccari, Diego Tomasi, Salvatore Filippo Di Gennaro, Jacopo Primicerio, Francesco Sabatini, Silvia Guidoni

**Affiliations:** 1 Istituto di Biometeorologia (CNR-IBIMET), Consiglio Nazionale delle Ricerche, Via G. Caproni 8, Firenze 50145, Italy; E-Mails: f.vaccari@ibimet.cnr.it (F.P.V.); f.digennaro@ibimet.cnr.it (S.F.D.G.); j.primicerio@ibimet.cnr.it (J.P.); f.sabatini@ibimet.cnr.it (F.S.); 2 Dipartimento Scienze Agrarie, Forestali e Alimentari, Università di Torino, Via Leonardo da Vinci 44, Grugliasco (TO) 10095, Italy; E-Mail: silvia.guidoni@unito.it; 3 Centro di Ricerca per la Viticoltura, Consiglio per la Ricerca e la Sperimentazione in Agricoltura, Conegliano (TV) 31015, Italy; E-Mail: diego.tomasi@entecra.it

**Keywords:** wireless sensor network, canopy microclimate, ZigBee, viticulture

## Abstract

A new wireless sensor network (WSN), called CrossVit, and based on MEMSIC products, has been tested for two growing seasons in two vineyards in Italy. The aims are to evaluate the monitoring performances of the new WSN directly in the vineyard and collect air temperature, air humidity and solar radiation data to support vineyard management practices. The WSN consists of various levels: the Master/Gateway level coordinates the WSN and performs data aggregation; the Farm/Server level takes care of storing data on a server, data processing and graphic rendering; Nodes level is based on a network of peripheral nodes consisting of a MDA300 sensor board and Iris module and equipped with thermistors for air temperature, photodiodes for global and diffuse solar radiation, and an HTM2500LF sensor for relative humidity. The communication levels are: WSN links between gateways and sensor nodes by ZigBee, and long-range GSM/GPRS links between gateways and the server farm level. The system was able to monitor the agrometeorological parameters in the vineyard: solar radiation, air temperature and air humidity, detecting the differences between the canopy treatments applied. The performance of CrossVit, in terms of monitoring and reliability of the system, have been evaluated considering: its handiness, cost-effective, non-invasive dimensions and low power consumption.

## 1. Introduction

Modern viticulture is one of the most promising agricultural sectors where new technologies might be implemented (*i.e.*, disease control, canopy management) to ensure high quality wine production, low management costs and good yield, to cope with the recent crisis in the sector. The International Organisation of Vine and Wine has recently published its report of the global economic situation of the viticulture and wine market in 2011, stating that the world vineyard area has decreased by 7.8 Mha (thousands of hectares). In the European Union the reduction in vineyard area could be between 50 and 55 Mha, with the largest reductions in Greece, Italy and Portugal. Quality is a critical factor for the success of wine and therefore strict control of the wine production process is of utmost importance [1].

Precision viticulture aims to maximize the oenological potential of vineyards. This is especially true in regions where the high quality standards of the wine production justify the adoption of site-specific management practices to simultaneously increase both quality and yield [2]. Furthermore, the site-specific management practices, which are becoming increasingly common, are also oriented towards a sustainable viticulture in which the growers and winemakers have begun to evaluate the impact of their management practices on the environment both for its preservation (*i.e.*, save water) and to characterize their product on the wine market [3].

Besides the prestige wines from historically suited environments, quality products can be obtained in Italy in sites appropriate for a modern and economically sustainable viticulture, such as the Veneto Piave CDO area. In these situations, mechanized vine management systems can support the competitiveness of wines able to satisfy a broad-based demand.

New Wireless Sensor Network (WSN) technologies can be useful and efficient to provide remote and real-time monitoring of variables involved in the grape and wine production system. In a broad sense a WSN is composed of various sensor modules connected to a node with radio modules that transmit the data from nodes to a base station where the data are stored.

A WSN integrates sensors, wireless communications, embedded computing, micro-electromechanical systems, microelectronics and other technologies; it can monitor parameters in real-time, sense and gather information from environments or objects, process the data and transmit the required information to the users.

The agricultural sector does not make sufficient use of technology and informatics to support production practices. Although progress has being made with regard to the deployment of sensors, WSN, actuators and other electromechanical devices in agricultural settings, there are still important areas of development that have not been sufficiently explored.

Embedded systems and wireless technologies can, in the long run, reduce costs and increase profits in countries with favorable year-round climates that permit multiple harvests but which lack other essentials required to maximize their potential [4].

The application of a WSN in viticulture might respond to the actual needs, although a complete cost and performance analysis of these networks is fundamental to fully understand their application potential. Indeed, the dimensions of a single sensor node may vary from the size of a shoebox to a microscopically small particle, while the cost of a single device may vary from hundreds of euros (for networks of very few but powerful nodes) to a few cents (for large-scale networks made up of very simple nodes) [5].

In the last years many system deployments and WSN architectures have been proposed and published in the scientific literature with different potential characteristics, especially with regard to precision farming applications, where energy efficiency, transmission performance as well as the effectiveness of the application have shown that these tools could make a contribution not only to research but also for the food industry. A comprehensive review on the state of the art of WSNs in agriculture and the food industry was written by Ruiz-Garcia *et al.* [6]. With regard to applications in viticulture, Burrell *et al.* [7] described WSN applications and configurations that can address different priorities in the vineyard, and Beckwith *et al.* [8] implemented a WSN consisting of 65 motes that collected temperature measurements in a vineyard over one month. Some of these applications focused on integration of WSN for video-surveillance. Lloret *et al.* [9] showed that, in the agriculture context, wireless technologies such as Wi-Fi and Bluetooth penalize energy consumption, becoming a major drawback when transmitting the images or video sequences required by an identification system. A video-surveillance platform called Integrated WSN Solution for Precision Agriculture was described and proposed by Garcia-Sanchez *et al.* [10] to detect and identify intruders as well as to oversee the production process. Very interesting studies have been conducted by the Portuguese CITAB and UTAD institutes. Researchers have shown the feasibility of a ZigBee-based WSN powered by batteries that can be recharged with green energy from three sources (solar power, wind power and hydro power) [11]. In addition, Peres *et al.* [12] developed the Intelligent Precision Agriculture Gateway (iPAGAT), designed to provide the necessary middleware between locally deployed sensor networks and a remote location within the whole-farm concept. This solar-powered infrastructure runs an aggregation engine that supplies a local database with environmental data gathered by a locally deployed ZigBee wireless sensor network. Finally, Nadimi *et al.* [13] have developed a WSN system with the same hardware described in our paper, although with totally different objectives.

This paper introduces a new WSN, called CrossVit, based on MEMSIC products and tested for two years in a vineyard in Northern Italy. The aims are to: (i) evaluate the monitoring performances of the new WSN directly in the vineyard; (ii) collect air temperature, air humidity and solar radiation data to support vineyard management, disease control and water management practices; (iii) compare vine canopy microclimate related to different training and trellis systems (*i.e.*, upwards or downwards shoot position) or canopy management practices (*i.e.*, leaf removal, shoot pruning, *etc.*).

## 2. Experimental Section

### 2.1. System Deployment and Architecture

**Figure 1.**
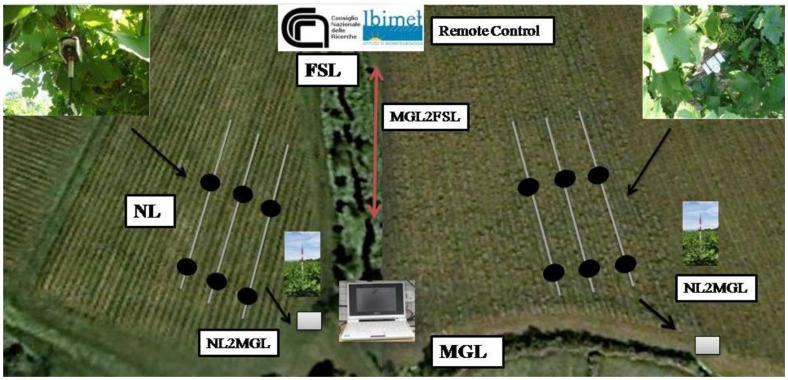
Illustration of the on-going implementation of the in-field data acquisition network. MGL = Master/Gateway Level; NL = Node Level; FSL = Farm Server Level; NL2MGL = Node Level to Master/Gateway Level connection; MGL2FSL = Master/Gateway Level to Farm Server Level connection; NL2MGL = Node Level to Master/Gateway Level connection.

The WSN system proposed is based on MEMSIC Technology devices and consists of three levels (Figure 1). The Master/Gateway level (MGL) coordinates the WSN and performs data aggregation. This level is associated with the Farm/Server level (FSL) that is responsible for storing data on a server, data processing and graphic rendering. The MGL is constituted by a gateway composed of a Mib520 and Iris device (Table 1) with a USB coupled to an Asus eeePC® connection and utilized as receiving station, data storage and node programming. The FSL is a remote Linux Server with a modem as receiving station. Nodes level (NL) is based on a network of peripheral nodes consisting of a MDA300sensor board and Iris module. The MDA300 board is a generic platform for measurements of analog and digital signals, with the option of providing a different power supply to the sensors.

The NL includes all the data collection procedures and is based on a WSN technology to acquire agrometeorological data inside the vineyard. The sensors installed on the system include thermistors for air temperature, photodiodes for global and diffuse solar radiation, and an HTM2500LF sensor (Humirel, Toulouse, France) for relative humidity. Wireless transmission between NL and MGL was obtained using two Iris ZigBee modules (Table 1), connected to a MIB520 (forming the gateway) and MDA300 (forming the node) respectively, with a frequency range of 2.4 GHz, IEEE 802.15.4 and about 100 m of coverage. In this scenario, levels of communication coexist: WSN links between gateways and sensor nodes by ZigBee (NL2MGL connections), and long-range GSM/GPRS links between gateways and the server farm level (MGL2FSL connections). This paper reports a WSN development based on a multi-level wireless network. The network consists of 12 nodes connected to three types of sensors (see Section 2.5: Sensors Equipment) that collect agrometeorological data within the canopy. Iris modules for transmission were programmed using the TinyOS 2 platform. This platform was chosen because it is a well-developed open source system and in addition, resources are available on the Internet for the development of applications based on it. The architecture of the implemented WSN is a star network topology where every node interacts only with the coordinator of the network localized in the center of the star. This is the simplest topology and allows the use of protocols and algorithms that are equally simple and easy to implement.

### 2.2. Wireless Communication

Among the available technologies for wireless communication: Wi-Fi (IEEE 802.11b), Bluetooth (IEEE 802.15.1), ZigBee (IEEE 802.15.4), the latter was chosen because it provides a better performance in terms of signal handling and cost. In fact, in recent years the majority of applications in precision agriculture include ZigBee technology as hardware. This technology ensures greater simplicity and lower costs than other technologies, including Bluetooth. Iris, developed by MEMSIC Technology, is the ZigBee module used in the system. The module characteristics are shown in Table 1.

### 2.3. Gateway

The gateway is a combination of three separate modules: a Mib520, a mini PC (Asus eeePC®) and a GSM/GPRS modem. The first module provides USB connectivity to the Iris and MICA motes, useful for communicating with a personal computer and programming. It supplies power to the device through the USB port. The programmer was provided with a processor, ATMEGA16L, necessary to program the motes. TinyOS operating system was installed in order to program the motes. The programmer is connected via USB to a computer eeePC with Xubuntu Linux version and TinyOS 2.1.0 installed. Audiotel GSM/GPRS modem is connected to the eeePC to ensure communication with the FSL.

The CrossVit gateway is powered by means of two 50 W peek solar panels combined with a rechargeable battery, under the control of a voltage supervision system. These solar panels charge two 12 V/100 Ah lead-acid batteries. The gateway is equipped with one weather proof quad-band GSM/GPRS antenna, equipped with a 3 m RG178 low drop signal cable (impedance 50 Ω) with max. attenuation of 0.3 dB, in order to ensure the MGL2FSL connections and one ZigBee antenna (2.4 GHz Omni RA7″ MMCX 5 dBi omni-directional antenna) for mote-gateway communication (NL2MGL connections) placed on a 3 m high post. The RJ-178B cable has a SMA male connector (antenna) and a male MMCX connector (mote).

### 2.4. Node

**Figure 2.**
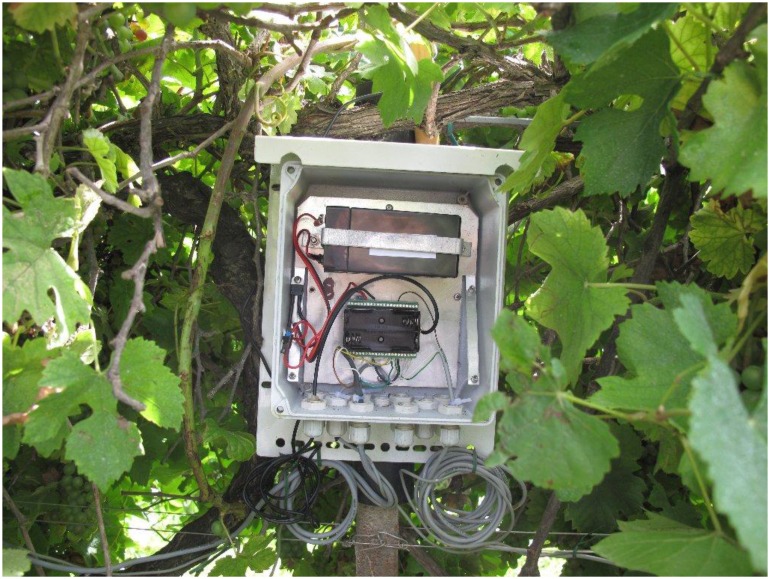
Node Level.

The nodes are composed of the Iris wireless module and MDA300 Sensorboard module. The MDA300 is a generic measurements platform for analog and digital data acquisition. Its characteristics are given in Table 1. The power supply consists of a 6 V/150 mA solar panels combined with a 6 V/4.5 Ah lead acid battery. A 5 V low-dropout DC/DC converter has been added to adjust the battery voltage. The elements were contained in a watertight case with IP 67 protection that was placed on a support inside the vine canopy, at the cordon level (Figure 2), while the solar panels were installed on a 2 m mast above the canopy.

### 2.5. Sensors Equipment

The wide availability of channels on the sensor board allowed the implementation of a system with a wide range of sensors (Table 2). Diffuse solar radiation, temperature and air humidity were the main parameters chosen for the aim of our study. After a detailed analysis of market products the node sensors that have shown better performance in terms of resolution, accuracy, response time, energy consumption and overall cost were selected.

The temperature is measured by two types of sensors placed inside a solar shield to avoid heating by incident light and provide shock protection. Both were positioned under the permanent vine row in order to monitor the microclimate inside the canopy. The first sensor is an epoxy-thermistor composed of a micro-BetaCHIP device 10 K 3MCD1 (Betatherm Sensors, Ballybrit Business Park, Galway, Ireland) protected by a plastic housing, which confers resistance to shock and moisture, without losing accuracy and speed of measurement. The second sensor is the HTM2500LF, a humidity and temperature transducer designed for monitoring applications where a reliable and accurate measurement is needed. The diffuse solar radiation sensor used in the system is the same as that employed on the NAV system. It is a prototype consisting of a silicon photocell in a PVC support under a Teflon dome, which can simulate the diffuse component of radiation. Each peripheral node is equipped with two radiation sensors, positioned inside the canopy below and just above the permanent vine cordon. The sensor does not require power supply, and provides an output signal as a continuous electrical voltage proportional to the amount of diffuse solar radiation. A calibration phase of each sensor used in the WSN was performed for a period of 10 days in experimental field conditions before system deployment in the vineyard. The wireless nodes were allocated on a support at 20 m distance from the master station, and the sensors were placed proximal to the reference sensors installed on a CR1000 datalogger (Campbell Scientific, Inc.CSI, North Logan, UT, USA). Calibration of the temperature sensor (thermistor) and temperature and humidity sensor (HTM2500LF) was done with the HMP45C (Campbell Scientific), a temperature and relative humidity probe containing a platinum resistance temperature detector (PRTD) and a capacitive relative humidity sensor. The solar radiation sensor (silicon photocell prototype) was calibrated with a global solar radiation sensor EPPLEY (mod. psps.n.20298F3, The EPPLEY Laboratory, Inc., Newport, RI, USA). Calibration was performed by acquiring data every 5 min for the duration of the calibration phase, and transmitting data from each node to the master station at hourly intervals.

### 2.6. Software Description and Integration

**Figure 3.**
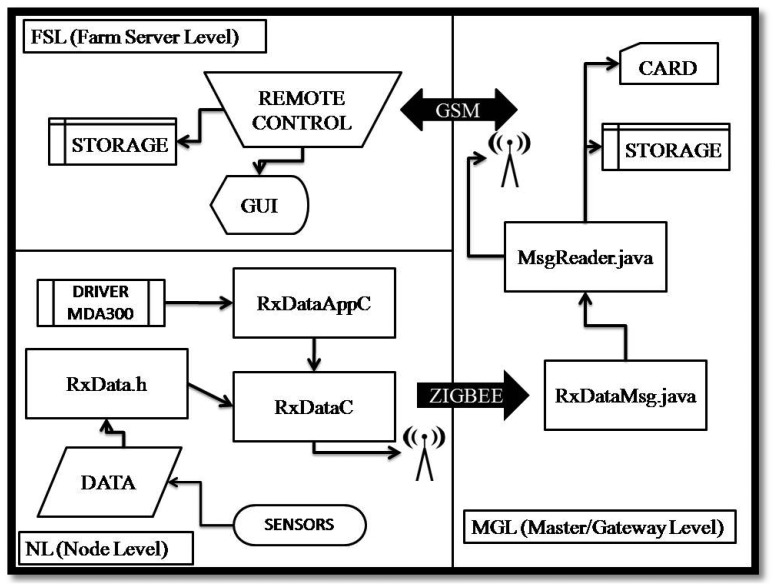
Block diagram of the hardware connections and software operation.

Network management was performed by software based on the TinyOS platform [14]. TinyOS is an open source operating system based on modules, designed specifically for WSN, developed by the nesC programming language. Figure 3 shows the block diagram of the hardware connections and software operation.

#### 2.6.1. NL Firmware

The sensor management program was operating in “split-phase” mode, which is a technique that realizes an asynchronous communication between components and is used to perform operation blockers of long duration, dividing the operation into two stages: (i) Invocation (command ends immediately) and (ii) Return the result (event notification). The sample events are controlled by a timer event Timer0.fired which calls an asynchronous event sensor reading (void Sensor_x_Read.readDone (error_t err, uint16_t value)). At the beginning of the sensor reading procedure, the radio module starts AMControl.start and once the packet is transmitted AMControl.stop. A Low Power Listening (LPL) mode is provided between reading sequences. With LPL, the nodes are woken up at periodic intervals called check intervals when they activate the radio and send the packet. Excitation commands were used for temperature and humidity sensors at 2.5 V and 5 V via the GeneralIO interface (e.g., instructions: GeneralIO as FIVE_VOLT) wired with the interface MicaBusC (e.g., instructions: App.FIVE_VOLT −> MicaBusC.PW5). The drivers used for the implementation of the sensor board MDA300 in the TinyOS were developed *ad*-*hoc* by Zheng *et al.* [15]. SensorMDA300ca is the configuration module which presents all interfaces. A total of eight single-ended AD channels, eight differential ended AD channels, and one digital IO channel are provided by this module. The command and event accomplished in any interfaces provided by SensorMDA300ca is the same. The command “read” can be invoked to start the AD converter. A “readDone” event will be triggered after the conversion is done. An interface from “sensorMDA300ca” has to be correctly wired in the application according to the hardware connection. The NL software consists of three integrated codes for the signals acquisition from the sensors and the transmission in packets form to MGL:

“RxAppAppC” was the component configuration defined by assembling existing components provided by TinyOS, it is a global configuration that defines the connections between all its components. “RxDataC” is the module that implements the interfaces exporting controls and event handlers. The application “RxDataC” needs to use four read interfaces aliased as “sensor_0_read” to “sensor_3_read”. In the corresponding configuration module, “RxDataAppC”, the four interfaces are wired to the interfaces provided by “sensorMDA300ca”. The interface number to be chosen from “sensorMDA300ca” is determined by the physical connection port a sensor is wired in. Each data packet that comes out of the mote contains several fields of data (uint 16). The data payload of the message is defined in “RxData.h”. The overall message format for the application is as follows

- Node ID (2 bytes)
- Counter (2 byte)
- Payload (20bytes):
- ADC data readings (8 readings of 2 bytes each)
- Battery voltage (2 bytes)

#### 2.6.2. MGL Firmware

The basic TinyOS utility application BaseStation (BaseStationP.nc, BaseStationC.nc) is used in order to receive data from the gateway. It acts as a bridge between the serial port and radio network. When it receives a packet from the serial port, it transmits it on the radio; when it receives a packet over the radio, it transmits it to the serial port. Because TinyOS has a toolchain for generating and sending packets to a mote over a serial port using a BaseStation, it allows PC tools to communicate directly with mote networks. This module was loaded into the Iris wireless module connected to Mib520. TinyOS also provides a set of APIs and tools to build applications that interact with the sensor network (API Java, Parser package). The “RxDataMsg.java” application is a class that implements a Java interface to the “RxDataMsg” and all the message specifications, for example the size. Finally, the application “Msgreader.java” is a method for the data management and reception of all messages, sent from the mote connected to the USB port. This method performs a series of operations when it receives a message of “DataMsg” type, containing the nodes sensor data:

(1) data conversion from count to mv (* 2.5/4096);
(2) data calibration using cross-calibration span and offset;
(3) printing out the packets coming from the mote on the Gateway;
(4) saving each package in an ASCII file.

#### 2.6.3. FSL Firmware

To access the MGL, equipped with a Linux Asus eeePc, a direct connection must first be established from the FSL. This is accomplished with a Point-to-Point Protocol (PPP) connection. On the Master side Mgetty was used, a versatile program to handle all aspects of a modem under Unix based systems. The Mgetty program opens a serial device, configures it appropriately, configures the modem, and waits for a connection to be made. When a connection is detected, Mgetty issues a login prompt, and then invokes the login program to handle the actual system login. At this point the user has complete control of the Master PC, can view log files to check for system integrity, access individual data files from every node, and even retrieve data files.

### 2.7. Field Experiment

The CrossVit system was tested in Veneto Region (North-East Italy) by the National Council for Agricultural Research CRA. The trial was conducted on two commercial vineyards (Ponte di Piave and San Nicolò) owned by Le Rive farm, located in the province of Treviso. In the first vineyard the Vitis vinifera variety was cv Pinot gris grafted on SO4, and in the second one Cabernet Sauvignon grafted on 1103 P, both were planted in 2002. Both vineyards, which are located on silty-clay soils, are trellis trained with free cordon with spur pruning and have an irrigation system. For both varieties two different methods of winter pruning have been used, which allow the comparison between three treatments:

- NP: no pruning;
- PM: mechanical pruning;
- PMa: manual pruning

For grape quality and health of bunches it is very important to study the varietal adaptability to different training systems and pruning methods, the two varieties were chosen for their skin color and habitus: high vigor and fast growth for Cabernet Sauvignon and low vigor for Pinot gris. The microclimate inside the canopy is one of the most useful parameters to study given its effect on grape color, sugar and aroma compounds accumulation. The vines were spaced 0.95 m and 1.3 m apart for Pinot gris and Cabernet Sauvignon respectively, while row spacing was 2.5 m for both varieties. The single wire was 1.5 m high and the shoots positioning was free because there were no other wires or catch wires above the permanent cordon

The trial was conducted over two growing seasons from April to September (2011 and 2012). The node sensors were positioned inside the vineyards, adopting an experimental layout representing the six treatments (two varieties and NP, PM and PMa pruning), with two replicates for each sensor and treatment (in total two main stations and six nodes each). The temperature, humidity and solar radiation sensors were positioned inside the canopy as follows:

- Temperature sensor just above the permanent cordon;
- Solar radiation sensor placed below (20 cm) the permanent cordon;
- Humidity sensors inside the canopy in the same position as the solar radiation sensor.

## 3. Results and Discussion

### 3.1. Power Management Performances

The energy consumption test, for both MGL and NL, was done in the laboratory (Table 3). The node performed the reading of the sensors (75 mA peak) every 15 minutes and sent data to the gateway (6 mA peak). The increased energy consumption in the reading phase was due to the 5 s. excitation needed by the HTM2500 sensors. The nodes were equipped with a full capacity 4,500 mA battery, therefore to estimate the available battery life, 60% of capacity was considered (3,000 mAh), giving an estimated autonomy of approximately 21 days. This autonomy appears to be optimal in a field application to ensure, in case of problems with solar charge, enough time to replace the batteries. Regarding the gateway, the Asus eeePC consumed 900 mA. Fortunately, in the field tests it was not necessary to replace batteries, thanks to the solar panel charge and the high number of sunny days during the summer period.

### 3.2. Data Transmission Performances

The transmission test was done in the laboratory before the field test using the Received Signal Strength Indication (RSSI) parameter and then directly in the vineyards to verify the signal and to map the vineyard to ensure a good displacement of the nodes. It is a measure of the signal power on the radio link, usually in dBm units, while a message is being received. It can be used for estimating node connectivity and node distance (although the relationship between distance and RSSI is noisy and not straightforward). Another use of RSSI is to sample the channel power when no node is transmitting to estimate the background noise, also known as noise floor.

**Figure 4.**
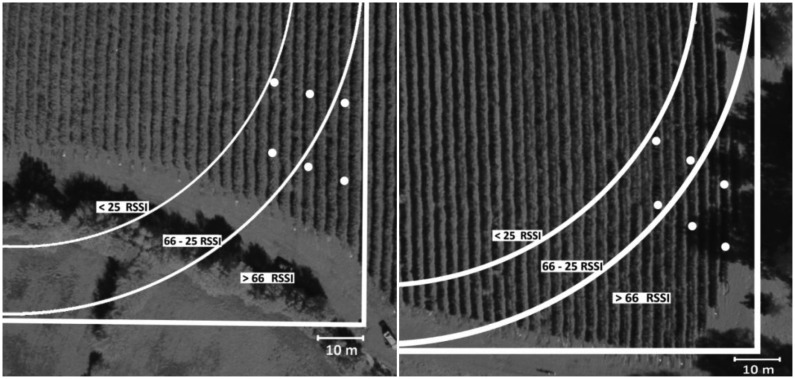
(**a**) Data transmission test at San Nicolò vineyard. The isosignal lines represent the RSSI values; (**b**) Data transmission test at Ponte di Piave vineyard. The isosignal lines represent the RSSI values.

The RSSI values given by TinyOS are usually not in dBm units, and should be converted by the platform-specific relationship to obtain meaningful data. In our field test we needed to have an indicator of the area with the minimum coverage sufficient for reception by the gateway of all packets sent by the nodes and not a parameter in absolute terms. The values range for RSSI values is 0–100, more distant, lower is this values, in fact, a high value means good packet reception, while a low value associated with a poor reception. The ping program sent the RSSI value continuously (every second) from the node connected to the gateway, and allowed a coverage map to be drawn of the network with RSSI levels. The isosignal lines associated with threshold values of RSSI and the position of the nodes are shown in Figure 4(a–b). All the nodes were then placed inside the area of >25 RSSI to allow a good data reception. The RSSI value measured in the field installation was not very high even at relatively short distances, due to the fact that the leaf canopy interfered with the transmission range. Tx errors were measured in terms of received data packages/sent data packages ratio resulting a value equal to 1 within the >25 RSSI value, while the ratio falls rapidly away from the 25 RSSI isosignal.

### 3.3. Agricultural Results

**Figure 5.**
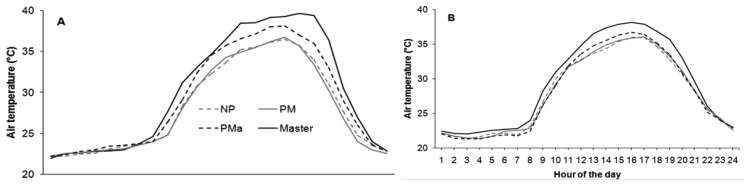
Daily cycle of air temperature (max) (°C) at the two vineyards A (Ponte Piave) and B (San Nicolò) in the three field treatments applied: NP, no pruning (gray dotted line); PM mechanical pruning (gray solid line); PMa manual pruning (black dotted line). Master (black solid line) is the air temperature measured outside the vineyard. All the values are hourly averages of two years of observations from April to September.

The field applications of CrossVit systems have shown good monitoring performance in both vineyards. The two systems were able to monitor the agrometeorological parameters in the vineyards: solar radiation, air temperature and air humidity, detecting the differences between the canopy treatments applied. In particular, the PMa treatment has shown the higher values of air temperature inside the canopy than the other two canopy management treatments in both vineyards, despite the different grapevine varieties having a typical trellis (Figure 5).

**Figure 6.**
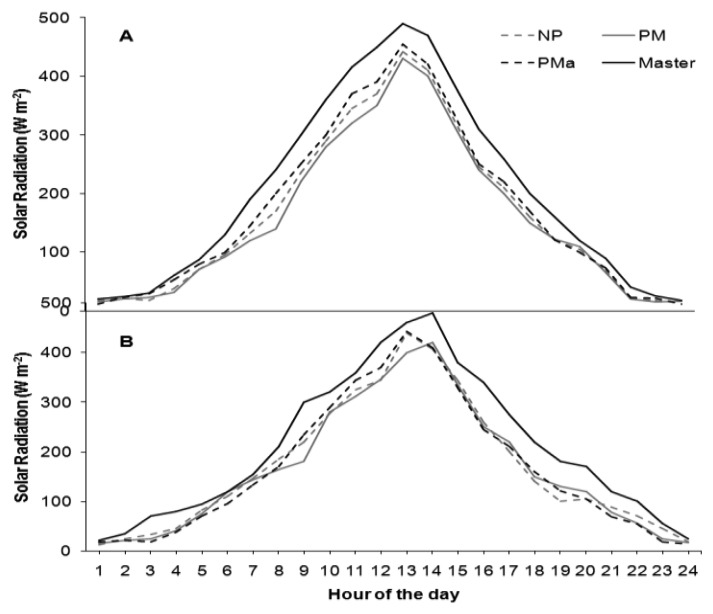
Daily cycle of solar radiation (W·m^−2^) at the two vineyards (**A**) (Ponte Piave) and (**B**) (San Nicolò) in the three field treatments applied: NP, no pruning (gray dotted line); PM mechanical pruning (gray solid line); PMa manual pruning (black dotted line). Master (black solid line) is the solar radiation measured outside the vineyard. All the values are hourly averages of two years of observations from April to September.

**Figure 7.**
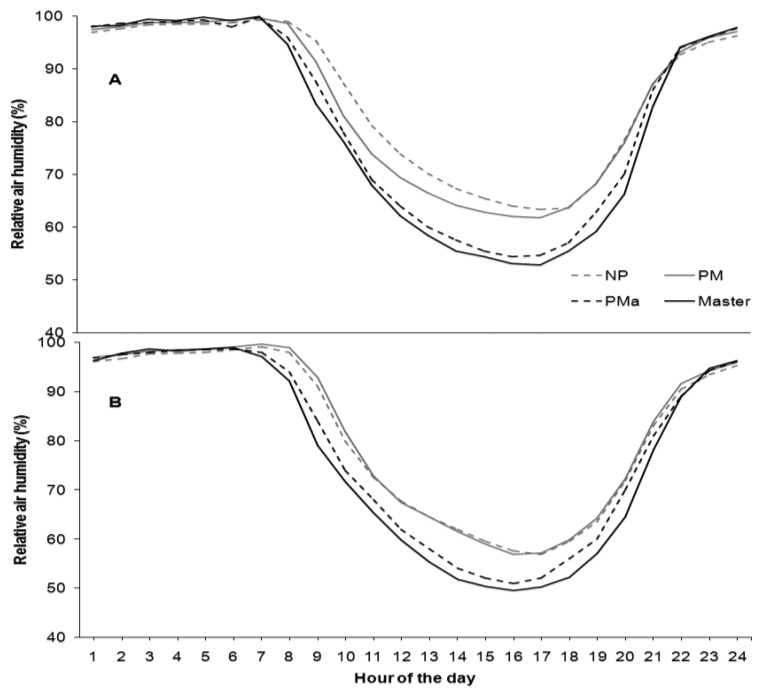
Daily cycle of relative air humidity (%) at the two vineyards (A) (Ponte Piave) and (B) (San Nicolò) in the three field treatments applied: NP, no pruning (gray dotted line); PM mechanical pruning (gray solid line); PMa manual pruning (black dotted line). Master (black solid line) is the relative humidity measured outside the vineyard. All the values are hourly averages of two years of observations from April to September.

As concerns solar radiation, the NP canopy management has been shown to receive less solar radiation in both sites (Figure 6). In accordance with these observations, the relative humidity in the PMa canopy was the lowest (Figure 7). In both sites and for all agrometeorological parameters, values recorded by the Master station were always higher compared with the Node values, consistently with the fact that the Master station was installed outside the vineyards without foliage coverage.

These two seasons' results indicated a satisfactory reliability of CrossVit systems in monitoring micrometeorological parameters and detecting the different canopy management effects. The CrossVit performance was evaluated considering the specific aims of this study:

1. Handiness: the solution had to be ready for use and easy to install; the installation was simply provided with two screws to the cordon pole.
2. Cost-effective: the cost of hardware, software and sensors had to be very low; the cost for a single complete node was approximately 300 euro.
3. Non-invasive dimensions: the nodes of the WSN had to permit the operations in the vineyard and not affect the surrounding environment; the case including the node and solar panel dimensions were 20 × 25 cm and 8 × 12 cm respectively.
4. Versatile and expandable: the system was developed for monitoring in the vineyard, but could be used for a wide range of applications and with the possibility of adding new types of sensors with small changes of the firmware (*i.e.*, irrigation schedule);
5. Low power consumption: the system should have low consumption in terms of energy supply and fully rechargeable with a small solar panel.
6. Firmware and software of the system must be developed in open source environments.

The CrossVit system is still being evaluated in the vineyards, but it has already been shown to have achieved many of the set goals. The results in terms of power supply and data transmission in the two seasons confirmed the reliability of the system. The agronomic results have shown the capability of CrossVit systems in monitoring micrometeorological parameters. However some weak points of the systems were identified, in particular, the high energy consumption of the receiving station data (Asus eeePc) and the quite complicated management software (TinyOS).

Regarding the first weak point, future development will be the implementation of the MGL based on an Arduino platform with the TinyOS operating system that promises a much lower energy consumption, even if having a PC in the field enabled the control of data supply chain and real time display, which was very useful. Studies on WSNs for agricultural application used different types of hardware and were often only aimed at a test run or concerned monitoring for few days in the field [13–16]. The present CrossVit system was tested in the vineyards for two seasons in analogy with the work of the Portuguese research team that started in 2008 [11] and then evolved as described in Peres *et al.* [12]. Our vineyards monitoring projects began in 2009 with the NAV system, which had good flexibility characteristics but weaknesses in terms of price and the proprietary software.

## 4. Conclusions

A new WSN application for real-time monitoring of vineyards is presented. The CrossVit system allowed two WSN to be implemented in two vineyards with different pruning treatments in order to understand whether the system could discriminate the effects of the treatments on the microclimate. This system has allowed us to collect data on the state of the crop with real time microclimate monitoring, not only for research purposes, but also for controlling events of management interest, such as early disease detection. The system was able to monitor the agrometeorological parameters in the vineyard: solar radiation, air temperature and air humidity, detecting the differences between the canopy treatments applied. This research has helped to demonstrate the feasibility of using WSNs for technical applications in precision viticulture. The methodology focuses on the monitoring of vineyards as a reference case but, with minor changes, it could be applied directly to other agricultural scenarios.

## Figures and Tables

**Table 1.** Hardware technical details.

IRIS module	MIB 520 module	MDA300 Sensor Board

Outdoor line-of-sighttests: 500m	Male to Female USB cable	7 single-ended or 3 differential ADC channels 10 bit
IEEE 802.15.4compliant RF transceiver	Baud Rate: 57.6 K	4 precise differential ADC channels
2.4 to2.48 GHz	Mo Interface Connectors: 51-pin	
Globally compatible ISM band	Jtag Interface	6 digital I/O channels with event detection interrupt
Resistant to RFinterference	Connector: 10-pin male header	
250 kbps data rate	USB Bus powered	2.5, 3.3, 5V sensor excitation and low-power mode
Pre-loaded with Mote Runner		64K EEPROM for onboard sensor calibration data
Provides inherent data security		External I2C interface

**Table 2.** Characteristics of the node sensors and relative on-board channels. Short sensors description, range, resolution, precision, operational temperature and power supply information are reported for each one.

**Channel #**	**A1**	**A2**	**A3**	**A4**	**A5**
Channel type	Analog	Analog	Analog	Analog	Analog
Parameter	Grape Radiation_1	Grape Radiation_2	Air Temperature_1	Air Temperature_2	Air Humidity
Sensor	Siliconphotocell	Siliconphotocell	Thermistor 10k 3MCD1	Thermistor 10k@25 °C	Humidity Sensor
Type	Prototype	Prototype	Sensor Scientific Epoxy-Coated Chip	HTM2500LF	HTM2500LF
Range	0÷1,400 Wm^−2^	0÷1,400 Wm^−2^	−30÷70 °C	−30÷85 °C	0÷100%
Resolution	0.1 Wm^−2^	0.1 Wm^−2^	0.1°C	0.1°C	0.1%
Precision	±5 Wm^−2^	±5 Wm^−2^	±1%	±1%	± 5%
Operation temperature	−20÷75 °C	−20÷75 °C	−30÷80 °C	−30÷80 °C	−30÷70 °C
Power Supply	-	-	2.5 V	5.0 V	5.0 V

**Table 3.** Energy consumption for node modules and gateway/master module. The table reports the total energy consumed in different duty cycles.

**Currents**		**Duty Cycles**	
	Value (mA)	%	mA-h
***Node*** (wireless module, sensor board and sensors)			
Logger	75	5	3.75
Transmission	6	5	0.3
Sleep	2	90	1.8
**Total**			**5.85**
***Gateway (Master):*** Asus eeePc with modem GSM/GPRS			
Idle	900	100	900
**Total**			**900**

## References

[1] (2013). Receuil des methods internationals d'analyse de vins et des moûts.

[2] Matese A., Di Gennaro S.F., Zaldei A., Genesio L., Vaccari F.P. (2009). A wireless sensor network for precision viticulture: The NAV system. Comput. Electron. Agric..

[3] Zucca G., Smith D.E., Mitry D.J. (2009). Sustainable viticulture and winery practices in California: What is it, and do customers care?. Int. J. Wine Res..

[4] Santos R.A., González-Potes A., Block A.E., Virgen-Ortiz R.A. (2011). Developing a new wireless sensor network platform and its application in precision agriculture. Sensors.

[5] Romer K., Mattern F. (2004). The design space of wireless sensor networks. IEEE Wirel. Commun..

[6] Ruiz-Garcia L., Lunadei L., Barreiro P., Robla J.I. (2009). A review of wireless sensor technologies and applications in agriculture and food industry: State of the art and current trends. Sensors.

[7] Burrell J., Brooke T., Beckwith R. (2004). Vineyard computing: Sensor networks in agricultural production. IEEE Pervasive Comput..

[8] Beckwith R., Teibel D., Bowen P. Report from the Field: Results from An Agricultural Wireless Sensor Network.

[9] Levis P., Madden S., Polastre J., Szewczyk R., Whitehouse K., Woo A., Gay D., Hill J., Welsh M., Brewer E. (2005b). TinyOS: An Operating System for Wireless Sensor Networks. Ambient Intelligence.

[10] Lloret J., Garcia M., Bri D., Sendra S. (2009). A wireless sensor network deployment for rural and forest fire detection and verification. Sensors.

[11] Garcia-Sanchez A.J., Garcia-Sanchez F., Garcia-Haro J. (2011). Wireless sensor network deployment for integrating video-surveillance and data-monitoring in precision agriculture over distributed crops. Comput. Electron. Agric..

[12] Morais R., Fernandes M.A., Matos S.G., Serôdio C., Ferreira P.J.S.G., Reis M.J.C.S. (2008). A ZigBee multi-powered wireless acquisition device for remote sensing applications in precision viticulture. Comput. Electron. Agric..

[13] Peres E., Fernandes M.A., Morais R., Cunha C.R., López J.A., Matos S.R., Ferreira P.J.S.G., Reis M.J.C.S. (2011). An autonomous intelligent gateway infrastructure for in-field processing in precision viticulture. Comput. Electron. Agric..

[14] Nadimi E.S., Jørgensen R.N., Blanes-Vidal V., Christensen S. (2012). Monitoring and classifying animal behavior using ZigBee-based mobile ad hoc wireless sensor networks and artificial neural networks. Comput. Electron. Agric..

[15] Zheng J., Elliott C., Dersingh A., Liscano R., Eklund M. Design of a Wireless Sensor Network from an Energy Management Perspective.

[16] Lopez J.A., Soto F., Suardiaz J., Sánchez P., Iborra A., Vera J.A. (2009). Wireless sensor networks for precision horticulture in Southern Spain. Comput. Electron. Agric..

